# The Domination of Penicillin G Degradation in Natural Surface Water: Effect of Calcium Ion and Biological Dissolved Organic Matter

**DOI:** 10.3390/antibiotics14111144

**Published:** 2025-11-11

**Authors:** Feng Sheng, Jingyi Ling, Na Mi, Jixing Wan, Lu Yang, Ming Li, Chao Wang, Jiaqi Shi

**Affiliations:** 1 Key Laboratory of Soil Environmental Management and Pollution Control, Nanjing Institute of Environmental Sciences, Ministry of Ecology and Environment, Nanjing 210042, China; shengfeng@nies.org (F.S.); mina@nies.org (N.M.); wanjixing@nies.org (J.W.); yanglu@nies.org (L.Y.); liming@nies.org (M.L.); 2 Jiangsu Province Engineering Research Center of Synergistic Control of Pollution and Carbon Emissions in Key Industries, Jiangsu Provincial Environmental Engineering Technology Co., Ltd., Nanjing 210019, China; chaowang@nju.edu.cn

**Keywords:** antibiotics, hydrolysis, photolysis

## Abstract

**Background:** Although hydrolysis and photolysis are important pathways for penicillin antibiotics degradation in aquatic ecosystems, the degradation mechanism of penicillin antibiotics in real natural waters is rarely reported. Furthermore, the dominant factors influencing this process are poorly understood. **Methods:** Therefore, five natural waters were selected to simulate both the hydrolysis and photolysis processes of penicillin G (PG) in aqueous environments. **Results:** Our results demonstrated that the half-life of PG hydrolysis ranged from 44 h to 141 h in natural water, and aqueous Ca^2+^ ion was the most important factor controlling the hydrolytic degradation of PG. Moreover, several biological dissolved organic matter (DOM, microbial by-product compounds) could also promote the PG hydrolysis reaction. Direct photolysis of PG is dominated in natural water, for which half-life photodegradation rates were 6 h in both blank and natural water, suggesting that salinity and DOM have little influence on penicillin photolysis. The hydrolysis reaction mainly involved the cleavage of the ester bond in the β-lactam ring and a decarboxylation process, while photolysis degradation principally included the hydroxylation of the benzene ring and destruction of the thiazole ring. **Conclusions:** This study demonstrates the significant factors influencing hydrolysis and photolysis of penicillin antibiotics in an aquatic ecosystem, which can improve the estimates of ecological risk of antibiotic pharmaceuticals in a realistic environment.

## 1. Introduction

Penicillin antibiotics are the longest-established and most globally utilized antibiotics [1]. Among them, penicillin G (PG) is the first antibiotic discovered by Alexander Fleming, which is most produced and widely used in medical treatments of humans and livestock nowadays [2,3]. For example, PG and other penicillin antibiotics account for 44% of the total usage of antibiotic medicine and 4.7% of the animal consumption from 2010 to 2011 in the U.S [4]. Due to the low portion of antibiotic consumption in human and animal bodies, a large amount of penicillin antibiotics is being released into the soil and water environment, leading to the environmental issues of superbugs and resistance genes [3,5,6]. Resistance genes have been proven to transform among environmental bacteria, which could be a potential threat to human and ecological health [7,8]. Therefore, investigating the migration and persistence of penicillin antibiotics in natural environments is crucial for predicting their environmental fate, assessing ecological and health risks, and formulating targeted pollution control strategies.

Although penicillin antibiotics have been widely utilized, their detected concentrations in wastewater, surface water, and seawater are typically as low as ~ng L^−1^ [9,10]. This low detection level is mainly attributed to the unstable β-lactam ring in penicillin molecules, and these factors easily induce hydrolysis or photolysis of penicillin molecules [11,12,13]. For instance, the hydrolysis rates of penicillin antibiotics in alkaline solution are higher than those in neutral conditions, since the hydroxide ions serve as a stronger nucleophile than H_2_O to catalyze the hydrolysis reaction. In addition, Mitchell found that hydrolysis rates of penicillin and cephalosporin antibiotics increased 2.5 to 3.9 fold for a 10 °C increase in temperature [14]. Moreover, penicillin hydrolysis might also be affected by ion strength [15], metal ions [16,17], metal oxide [18], and clay minerals [19]. However, most existing studies have focused on hydrolysis under controlled laboratory conditions, while research on penicillin hydrolysis and photolysis in real natural water bodies, which contain complex matrices, is still scarce. The dominant mechanism of penicillin antibiotic degradation in natural water bodies is essential for risk assessment of environmental resistance genes, which is rarely reported. The knowledge gap makes it difficult to accurately evaluate the actual environmental persistence and potential ecological risks of penicillin antibiotics in natural aquatic systems.

The objective of this study is to investigate the dominant parameters affecting hydrolysis of penicillin antibiotics in realistic waters. The natural water samples were investigated in hydrolysis and photolysis experiments by creating complicated and realistic reaction conditions, which are different from previous studies. PG, as the most widely used penicillin antibiotic, is selected as the target antibiotic, and five surface waters are collected to simulate the hydrolysis and photolysis process. Hydrolysis kinetics rates (k_obs_) and HPLC-QTOF-MS-MS were adopted to analyze the main factors and reaction products, respectively. This study aims to provide fundamental insights into the abiotic degradation processes of penicillin antibiotics, which supports environmental risk assessments of these compounds in aquatic ecosystems.

## 2. Results and Discussion

### 2.1. PG Hydrolysis in Different Surface Waters

Based on the physicochemical parameters of five water samples (Table S1), the pH values of all these waters ranged from 7.8 to 8.5, and the concentrations of dissolved organic matter (DOM) were in the range between 5 and 10 mgC L^−1^. Their concentrations of cations and anions were different, with the salinity in XWL being the lowest. Different hydrolysis rates (k_obs_, h^−1^) of PG in the five surface waters have been detailed in Table 1. The hydrolysis rates ranged from 0.0049 h^−1^ to 0.0157 h^−1^, and the half-life of the degradation rate varied from 44 h to 144 h. Specifically, the hydrolysis rate of PG in YSL is three times higher than that in XWL, suggesting that different compositions in water had a significant influence on PG degradation. However, the hydrolysis rates in all the waters are apparently higher than that in the simulated PBS solution (k_obs_ = 0.0024 h^−1^), indicating that specific components such as metal cations, anion ions, and organic matter might play significant roles in PG hydrolysis. According to the results of Mitchell, pH was considered the most important parameter in controlling the hydrolysis rates of penicillin and cephalosporin antibiotics [13]. Therefore, PG hydrolysis at different pH levels was first conducted in the YSL system (Figure S1). As a consequence, the hydrolysis rate of PG increased with increasing pH, but decreased at pH above 8.5 (Figure S1). It is in contradiction with the previous studies that hydrolysis rates of penicillins or cephalosporins in a simulated system would increase extremely when the solution pH was over 8 [13,20]. We speculated that pH might not be the dominant factor in PG hydrolysis in natural water bodies, since pH was determined by both properties and concentrations of different cations, DOM, and anions (especially carbonate). Therefore, to further confirm the effects of aqueous components on hydrolysis, correlation analysis, treated samples (C column and Na column), and additional cations (Ca, Mg, heavy metals, etc.), experiments were conducted.

### 2.2. Effects of Aquatic Parameters on PG Hydrolysis

A correlation between k_obs_ and properties of surface water (salinity, cations and anions, TOC, etc.) was established. The results in Table 2 show that the parameters, including conductivity, SO_4_^2−^, Ca, Mg, Total S, and Mn, significantly correlated with hydrolysis rates (k_obs_) in five natural waters, while others did not (*p* > 0.05). Among them, the concentrations of Mn exhibited a negative correlation with k_obs_, indicating that aqueous Mn might not be a significant driver in PG hydrolysis. In general, PG hydrolysis rate in natural water might be predominantly related to the free cation ions, including Ca and Mg. Previous studies demonstrated heavy metal ions (e.g., Cu, Zn) [16,17] could promote PG hydrolysis, while Cu, Zn, and other heavy metals have no significant correlation with k_obs_ in our experiment. This discrepancy might be explained by different concentrations between cation ions (Ca, Mg, etc.) and heavy metal ions (Cu, Zn, etc.). Specifically, the concentration of Ca and Mg ranged among ~mg L^−1^, while concentration of heavy metals ranged among ~μg L^−1^, which were a thousand times lower than cation ions. Due to their higher concentration, cation ions could compete for coordination sites against heavy metal ions, with PG to serve as the dominant catalytic hydrolysis.

**Figure 1:**
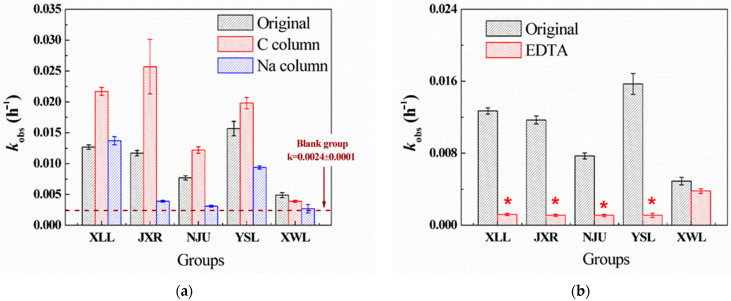

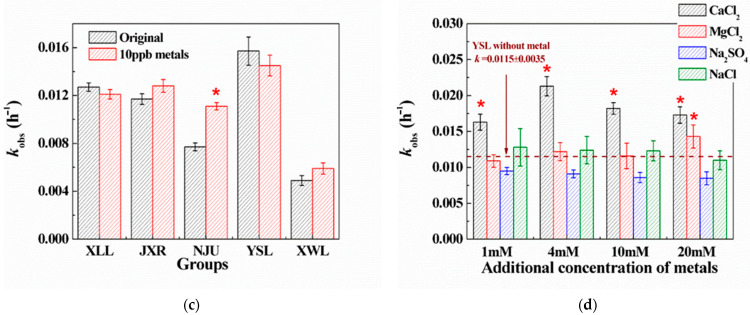
Degradation kinetics constants (k_obs_, h^−1^) of PG hydrolysis in the five surface waters with different treatments. (**a**) PG hydrolysis in the original, C column-, and Na column-treated samples. (**b**) PG hydrolysis in the original and EDTA-treated (1 mM) samples. (**c**) PG hydrolysis in five surface waters with additional heavy metals, including Fe, Zn, Cu, Mn, Co, Ni, and Pb (10 μg L^−1^). (**d**) PG hydrolysis in YSL water with the additional CaCl_2_, MgCl_2_, Na_2_SO_4_, and NaCl (1, 4, 10, and 20 mM). The initial concentration of PG was 20 μM, and the temperature of the reaction was 30 °C. * means a significant correlation (*p* < 0.05) against other groups.

Five sampled waters were filtered through the C column and Na column to eliminate the components of organic matter and cation ions (except for Na), respectively. After Na column treatment, most cation ions in the five waters were replaced by Na ions, and the concentrations of most heavy metals (Al, Fe, Mn, etc.) were undetectable (Table S2). On the contrary, the concentrations of Ca^2+^, Mg^2+^, and K^+^ in the C column treatment were nearly unchanged compared to the original waters, and the TOC concentration of these samples was all below 1 mg L^−1^. According to Figure 1a, lack of cation ions in the Na column treatment inhibited the PG hydrolysis, while the organic matter removal in the C column treatment facilitated PG hydrolysis. Thus, cation ions (Ca, Mg, and heavy metals) were deemed to be a significant factor in the hydrolysis of PG. Additional EDTA (1 mM) was added to further confirm the effects of cations on hydrolysis. The dominant inhibition was observed in four waters (XLL, JXR, NJU, and YSL, Figure 1b), suggesting the important role of natural cations. Among them, there was no significant difference in the original, C column, and Na column XWL (Figure 1a), probably due to the relatively low salinity in XWL. The hydrolysis rate of PG in XWL was also the lowest, indicating that cation-catalyzed hydrolysis might not be the main pathway of PG hydrolysis in XWL water.

Many previous studies demonstrated that the existence of heavy metals like Fe^3+^, Cu^2+^, Zn^2+^, Mn^2+^, etc., could promote the hydrolysis rates of PG by the formation of metal-PG complex [4,7,21,22,23]. In five waters, the concentration of heavy metals ranged from 1 to 10 μg L^−1^; therefore, the additional mixing of metal solution (containing Fe, Zn, Cu, Mn, Co, Ni, and Pb, 10 μg L^−1^) was spiked in the natural water to further confirm the role of heavy metals. Compared with the hydrolysis rates of PG in the original water, there was no significant difference except for that in NJU (Figure 1c). Since the concentration of heavy metals all ranged ~μg L^−1^, which is thousands of times lower than DOM (~mg L^−1^) and alkaline metals (Na, Mg, Ca, K, ~mg L^−1^), the competition complex between PG and alkaline metals against heavy metals inhibits their catalytic ability for PG hydrolysis. However, the detected concentration of Fe (1869 μg L^−1^) in NJU was much higher than others, and the slight increase of k_obs_ was observed in NJU, indicating that the Fe ion in NJU could partly participate in PG hydrolysis when its concentration was as high as ~mg L^−1^. Combined with correlation analysis, we added different concentrations of cations (NaCl, CaCl_2_, MgCl_2_, and Na_2_SO_4_) in YSL to estimate the effects of Ca, Mg, SO_4_^2−^, and salinity. Figure 1d demonstrates that the hydrolysis rates (k_obs_) increased with the increasing concentration of Ca^2+^, while others did not. It suggests that Ca^2+^ might coordinate with PG to form a Ca-PG complex to accelerate the hydrolysis reaction, and Mg, SO_4_^2−^, and salinity had little effect on degradation.

After the treatment of the C column, hydrolysis rates of PG generally increased compared to the original groups. For example, k_obs_ (0.024 h^−1^) of PG in the C column-treated JXR was twice more than that of the original JXR (k_obs_ = 0.012 h^−1^). We speculated that different types of DOM in natural water might contribute to PG hydrolysis, since not all the DOM could be removed by the C column. Therefore, the fluorescence spectra of five different waters filtrated by the C column and Na column were obtained, accompanied by the original sample. As illustrated in Figure S2, the types of DOM in the five waters were different, mainly containing the fulvic (Region III) and humic acid-like materials (Region V). However, after the C column treatment, the decreased intensity in Regions III and V suggested that most of the fulvic and humic acid-like materials have been extracted. On the contrary, the intensity of soluble microbial byproduct-like materials in Region III remained high, especially in the JXR and YSL samples, indicating that soluble microbial byproduct-like materials, such as protein, amino acid, and glucose, might participate in the hydrolysis reaction. Therefore, the degradation kinetics constant of PG hydrolysis in YSL with the additional tryptophan, albumin, phenylalanine, cysteine, and glucose as the characterized microbial organic matters was investigated. Among them, additional tryptophan, albumin, and cysteine showed the acceleration in PG hydrolysis, while phenylalanine and glucose did not (Figure S3). We deduced that the low-molecular-weight organic matter, especially amino acid molecules, could catalyze the PG hydrolysis in natural water. Llinás and Morris’s studies also demonstrated that through the formation of a reaction intermediate, thiols and amines could catalyze the hydrolysis of benzylpenicillin [24,25]. The amide molecules generated from microbial by-product compounds (like cysteine, tryptophan, etc.) might somewhat promote the PG hydrolysis, since the concentration of the microbial by-product compounds was much less than fulvic and humic acid in natural water. In general, the enhanced hydrolysis rate of PG was dominantly caused by the effects of Ca ion and microbial by-product compounds in natural water. The complex between Ca and PG to form a reaction intermediate and the amino acid or thiols acting as strong nucleophiles would synergistically catalyze the PG hydrolysis in natural environments.

### 2.3. PG Photolysis in Different Surface Waters

**Figure 2:**
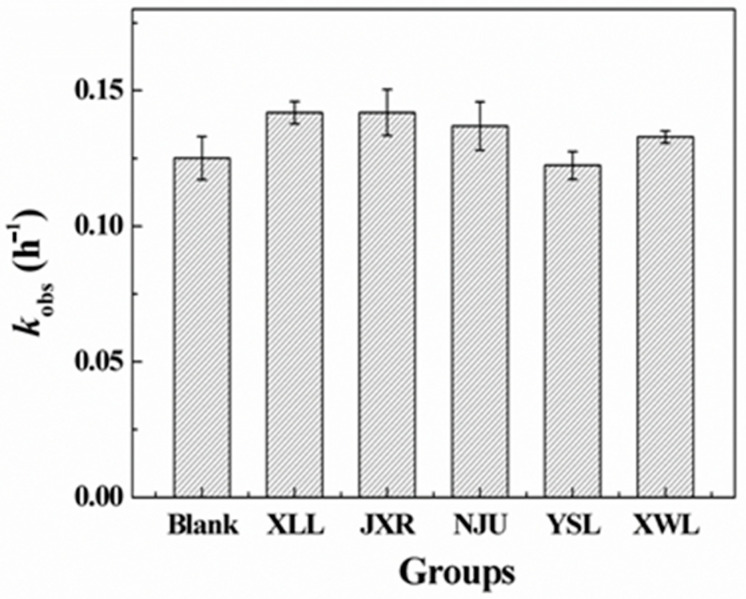
Degradation kinetics constants (k_obs_, h^−1^) of PG photolysis in five surface waters. Initial concentration of PG was 20 μM. Blank group refers to the photolysis of PG in PBS buffer (10 mM, pH = 8) under the same irradiation conditions.

Photodegradation of penicillins was also a main degradation pathway in natural rivers, lakes, and ponds [26,27,28]; hence, photolysis reactions of PG in both the PBS solution and five natural waters were conducted. As indicated in Figure 2, the photodegradation rates of PG in the five water samples showed no significant difference compared to the blank control, indicating that variations in dissolved organic matter (DOM) and metal content among natural waters had negligible effects on indirect photolysis processes. Therefore, the direct photodegradation was the dominant pathway for PG in natural environments. However, the apparent difference of k_obs_ was also observed (Figure 2), which might be caused by the different shield effects of DOM in waters.

The complexity of photolysis in the presence of natural organic matter has been introduced in previous studies [29]. Specifically, the DOM-photosensitized effects, including ROS (reactive oxygen species), light absorption and scattering, and adsorption, would also influence the photolytic degradation process of antibiotics in the aquatic environment. In addition, photodegradation of amoxicillin by the excited DOM (3DOM*) was also investigated to be the dominant route in natural organic matter isolate solutions [27]. However, direct photolysis of PG was confirmed to be the main degradation pathway in this study, and indirect reactions initiated by ROS or adsorption could be neglected, because high concentrations of anion ions could compete for ROS, and cation ions might be devoted to complex with PG molecules for stabilization in solution.

### 2.4. Product Analysis

The hydrolytic and photolytic products of PG in natural water were investigated by the LC-QTOF-MS-MS analysis. The products of PG hydrolysis were first detected and summarized in Table S3. As a result, we can see that the peak of PG appears at 20.02 min, and the main hydrolysis products, including penicilloic acid, benzylpenilloic acid, and 6-aminopenicillanic acid, were obtained. The fragment ions of penicilloic acids were similar at 17.79 and 18.02 min in chromatograms, indicating that the two obtained products of penicilloic acids were the chiral molecules. Similarly, two chiral products of benzylpenilloic acid and 6-aminopenicillanic acid were also confirmed. In addition, the presence of peaks at the retention times (RT = 22.83 and 24.36 min) was regarded as the hydrolysis product 5 (2-(amino(carboxy)methyl)-5,5-dimethylthiazolidine-4-carboxylic acid) and 6 (5,5-dimethylthiazolidine-4-carboxylic acid), respectively (Table S3). The detected products of PG hydrolysis in this experiment were also proposed in previous studies. For example, Hirte also found that different diastereomers of AMX penicilloic acids and AMX penilloic acids were detected, of which structures were similar to the penicilloic acid (TP 1) and benzylpenilloic acid (TP 2) [20].

**Figure 3:**
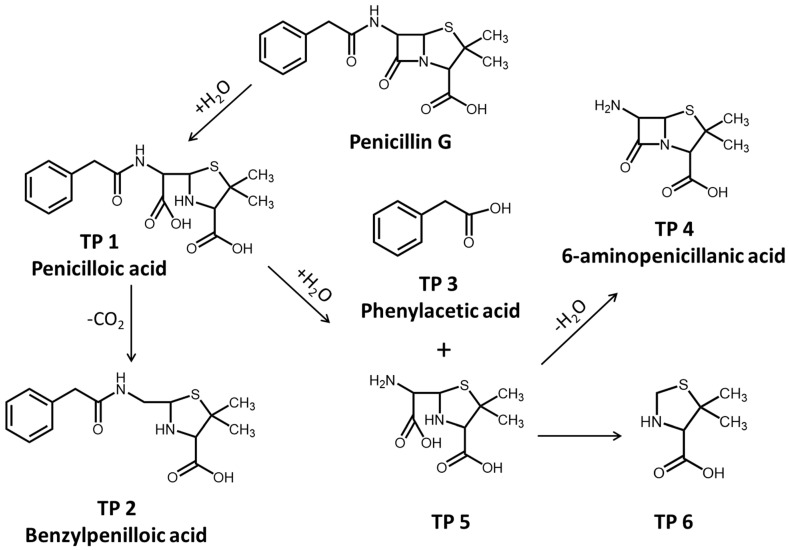
Hydrolysis pathway of PG in natural water.

In brief, the reaction pathways for PG hydrolysis were initiated by the cleavage of the ester bond in the β-lactam ring, and then decarboxylated to form benzylpenilloic acid (Figure 3). The active sites of PG hydrolysis could also occur in the branched amide bond to form TP 4 and TP 5. Furthermore, the branched chain in TP 5 could continue to hydrolyze to form TP 6. Penicilloic acid and benzylpenilloic acid were detected as mainly hydrolysis products in our experiments, which were found to be relatively environmentally persistent in natural water [1]. These degradation product residues in the environment might reveal the potential environmental risks, which should be evaluated in the future.

**Figure 4:**
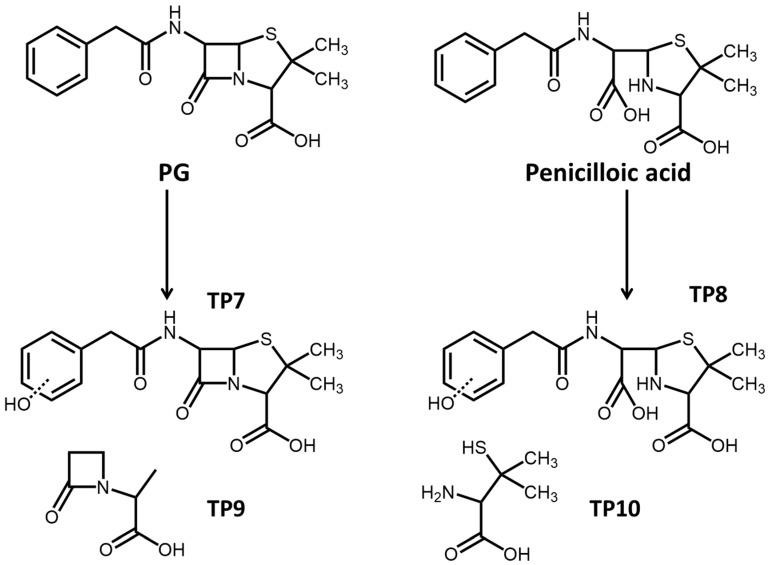
Photolysis pathway of PG in natural water.

Photolytic products of PG were also measured, and hydrolysis products like penicilloic acid could also be found in the photolysis process. The primary photolytic products involved the hydroxylation of PG and penicilloic acid, which dominantly occurred in the benzene ring to form the intermediate products (TP 7 and TP 8, Figure 4). In the direct photolysis process, the thiazole ring in the PG molecule would be destroyed to form low-molecular-weight carboxylic acids as TP 9 and TP 10 (Figure 4). Furthermore, volatile products, including benzene and toluene, were also found during the PG photolysis. The low-molecular-weight products were mostly volatile substances and not stable in the environment, with ecological risk being relatively low.

### 2.5. DFT Calculation

**Figure 5:**
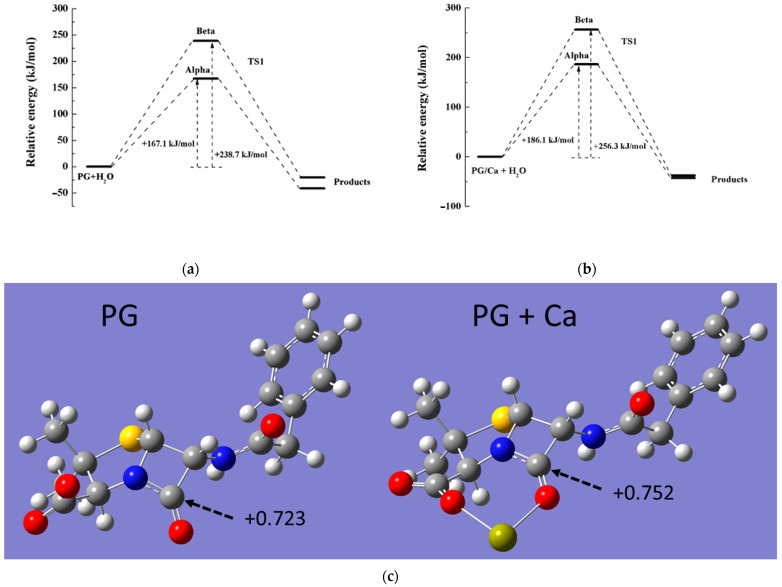
Calculated energy of transition state in (**a**) direct hydrolysis of PG molecule and (**b**) hydrolysis of PG-Ca complex from alpha or beta-side attack. (**c**) Optimized molecular geometries of PG and PG-Ca complex molecules.

To further confirm the hydrolysis process in the Ca-PG complex, theoretical computation was used to identify the reaction pathway and the associated energy of the transition state (TS). Since the carboxyl group of the PG molecule would be completely deprotonated in neutral solution, the PG species was selected as a target model in the calculation. Previous research demonstrated that the Ca^2+^ ion could be coordinated either via the O atom in carboxyl and the O atom in carbonyl in beta-lactam of the PG molecule [30]. Therefore, the structure of the Ca-PG complex has been optimized by using the basis set of 6-311G(d,p), and the optimized PG molecule was also obtained in Figure 5c. After the Ca^2+^ ion coordination, the positive charge of carbocation in the β-lactam ring increased from +0.723 to +0.752 (NBO charge, Figure 5c), indicating that the Ca-PG complex was more likely to be attacked by nucleophilic reagents in hydrolysis. Due to the spatial structure of the PG molecule, PG hydrolysis could be attacked by H_2_O from different directions (alpha-side and beta-side, Figure S4). During the hydrolysis, H_2_O attacked the amido bond in the β-lactam ring to form the TS, which was structured with H_2_O and the amide group from both alpha and beta sides (Figure S5). Based on the different activation energies of PG or PG/Ca hydrolysis, the activation energies of both PG and PG/Ca hydrolysis from alpha-side attack were lower than those from beta-side attack, indicating that H_2_O preferred to attack the amido bond from alpha-side (Figure 5a,b). However, the activation energy of Ca-PG (186.1 kJ mol^−1^) was higher than that of PG (167.1 kJ mol^−1^) itself, which did not correspond with the degradation rates in PG and PG/Ca hydrolysis. We speculated that direct hydrolysis catalyzed by H_2_O might not be the dominant pathway of PG in the hydrolysis of the Ca-PG complex. The hydroxyls combined with the Ca^2+^ ion might act as a strong nucleophile to attack the carbocation in β-lactam, which contributed to the rapid hydrolysis in the Ca-PG complex. Huang et al. also found similar catalyzed mechanisms of penicillin and cephalosporin antibiotics by metal ions, including Cu, Fe, Zn, and Mn [4,21,22,31,32,33].

## 3. Materials and Methods

### 3.1. Chemicals

Penicillin G sodium (PG, 98%), *L*-phenylalanine (98%), *L*-tryptophan (99%), *L*-cysteine (98%), *D*-glucose (97%), and albumin (Bovine) were all purchased from J&K Chemicals (Shanghai, China) without further purification. Sodium azide (NaN_3_), hydrochloric acid (HCl), sodium ethylene diamine tetraacetic (EDTA), sodium hydroxide (NaOH), sodium sulfate (Na_2_SO_4_), sodium chloride (NaCl), magnesium chloride hexahydrate (MgCl_2_ 6H_2_O), phosphoric acid, calcium chloride (CaCl_2_), and zinc nitrate hexahydrate (Zn(NO_3_)_2_ 6H_2_O) were purchased from Sinopharm Chemical Reagents (Shanghai, China). Methanol (HPLC grade), acetonitrile (HPLC grade), and formic acid (HPLC grade) were obtained from Sigma-Aldrich (Saint Louis, MO, USA) and used as received. Milli-Q water (18.2 MΩ cm) was used after being filtered through a 0.45 µm membrane in the experiments.

### 3.2. Sampling of Natural Water

Surface water (5–15 cm) was collected at the center of five lakes and rivers, including Xianlin Lake (XLL, 32°07′33″ N 118°59′16″ E), Jiuxiang River (JXR, 32°06′39″ N 118°57′01″ E), Nanjing University Pond (NJU, 32°07′09″ N 118°57′13″ E), Yangshan Lake (YSL, 32°06′28″ N 118°56′37″ E), and Xuanwu Lake (XWL, 32°04′56″ N 118°47′32″ E) in Nanjing (Figure S6). The samples were stored in the refrigerator at 4 °C until use within 7 d. The basic physical and chemical parameters of five water samples involving pH, total organic carbon (TOC), conductivity, concentration of anions (Cl^−^, NO_3_^−^, and SO_4_^2−^), concentration of cations (K^+^, Ca^2+^, Na^+^, Mg^2+^, and heavy metals), concentration of total S and P were determined in Table S1 and their detection methods were shown in Table S2.

### 3.3. Analysis Method

Concentration of PG was determined by HPLC analysis (Waters 2695, Milford, MA, USA) equipped with a diode array UV detector. Separation was carried out by using an Atlantic T3 C18 column (Waters, 5 μm, 4.6 × 250 mm), and the mobile phase contained 65% A phase (0.1% H_3_PO_4_) and 35% C phase (acetonitrile) with a detection wavelength of 210 nm. A flow rate of mobile phase was 1 mL min^−1^, and the column oven and the injection volume were set as 35 °C and 40 µL, respectively. The hydrolysis products of PG were determined by a high-performance liquid chromatography coupled with a high-resolution hybrid quadrupole time-of-flight mass spectrometer (HPLC-QTOF-MS-MS), and detailed information is shown in Content S1.

The physicochemical parameters of surface water, including pH, conductivity, concentrations of cation and anion ions, total P, total S, and total organic carbon, were also detected, and detailed methods are described in Content S2. Fluorescence excitation-emission matrix (EEM) spectrophotometry was utilized to characterize the natural dissolved organic matter (DOM) [34], and further information is observed in Content S3.

### 3.4. Hydrolysis and Photolysis of PG in Surface Water

The surface water (20 mL) was filtered through a 0.45 μm sterile membrane filter (Cellulose nitrate, Whatman, Maidstone, UK) and spiked with 1 mM NaN_3_ to inhibit the biological activities in 30 mL bottles. These bottles were incubated in a water bath at 30.0 ± 0.5 °C. 80 μL PG solution (5 mM) was added to initiate the reaction in the dark, and aliquots of 1 mL solution were sampled every day by adding EDTA (0.2 M, 10 μL) solution. Then the concentration of PG was analyzed by HPLC within 24 h. Photolysis experiment of PG in surface water was conducted in a photochemical reactor (XPA-7, Xujiang Electromechanical INC., Nanjing, China) equipped with a 500 W xenon lamp. The 280 nm filters were positioned to simulate solar light irradiation. The initial concentration of PG was 50 μM, and 1 mL sample was collected at certain intervals. All the experiments were conducted in duplicate. Analysis of variance was conducted using the ANOVA model. Significant difference (*p* < 0.05) was determined based on Tukey’s multiple-rate test. The relationship between k_obs_ and water properties (pH, conductivity, Cl^−^, NO_3_^−^, SO_4_^2−^, K, Ca, Na, Mg, Ba, total P, total S, Al, Mn, Co, Ni, Cu, Zn, Fe, and TOC) was evaluated in a stepwise multiple regression analysis. The degradation kinetics of penicillin were modeled by the pseudo-first-order kinetic Equation (1). Where C_t_ is the concentration of penicillin (µM), k_obs_ is the degradation constant of penicillin (h^−1^). Equation (1) can then be transformed into Equation (2), and the value of k_obs_ for penicillin degradation could be obtained by fitting the variation in penicillin concentration (µM) vs. reaction time (h), where C_0_ and C_t_ represent the initial concentration (µM) and the concentration of penicillin (µM) at the interval time t (h), respectively. The half-life degradation rate (T_1/2_, h) was calculated as Equation (3).

(1) $$dC_{t}/dt=-k_{obs}\times C_{t}$$

(2) $$lnC_{t}=lnC_{0}-k_{obs}\times t$$

(3) $$T_{1/2}=ln2/k_{obs}$$

### 3.5. Density Functional Theory (DFT) Calculation

DFT calculation of the energy barrier for PG hydrolysis was performed by the Gaussian 09 package. Geometry optimization of (protonated) PG molecules in the ground state was obtained using both B3LYP/6-31G(d,p) and B3LYP/6-311G(d,p) levels. The transition state (TS) of PG hydrolysis was identified by following the different directions (α-side and β-side) attacking from one water molecule to the β-lactam bond of PG, which was achieved using the same method and basis set for ground-state optimization. The integral equation formalism of the polarized continuum model (IEEPCM) was applied to simulate the hydrolysis process of PG in solution.

## 4. Conclusions

Hydrolysis and photolysis are important abiotic pathways for penicillin antibiotics in natural water. Our results reveal that Ca^2+^ ions and microbial by-product compounds in DOM, like cysteine, can promote the PG hydrolysis in natural environments. Specifically, aqueous Ca^2+^ ions can coordinate with the active functional groups in PG to form a complex, and Ca-bound hydroxyls act as the strong nucleophile to attack the amide bonds. Meanwhile, the presence of microbial by-product compounds, including cysteine, tryptophan, and albumin, can also catalyze the thiolysis and aminolysis of PG. The hydrolysis products of PG contain penicilloic acid, benzylpenilloic acid, and 6-aminopenicillanic acid, etc. The direct photolysis of PG in five natural waters has also been confirmed, and degradation products mainly involve the hydroxylation of PG and low-molecular-weight carboxylic acids. Our study reveals the dominant factors in the hydrolysis of PG and penicillin antibiotics in natural environments, which provides significant evidence for the assessment of the environmental risk of penicillin antibiotics.

## Figures and Tables

**Table 1:** Pseudo-first-order kinetics fitting of PG degradation in five natural waters.

Location	Fitting Equation	R^2^	k_obs_ (h^−1^)	T_1/2_ (h)
XXL	y = −0.0127x + 0.0444	0.99	0.0127	55
JXR	y = −0.0117x + 0.0669	0.99	0.0117	59
NJU	y = −0.0077x + 0.0315	0.99	0.0077	90
YSL	y = −0.0157x + 0.0746	0.98	0.0157	44
XWL	y = −0.0049x + 0.0344	0.98	0.0049	141
Blank	y = −0.0024x − 0.0151	0.99	0.0024	289

**Table 2:** Correlation between k_obs_ and physicochemical properties of natural water.

Parameter	Correlation Coefficient	Significance (*p*)
pH	−0.483	0.410
Conductivity	0.906	0.034 *
Cl^−^	−0.304	0.619
NO_3_^−^	−0.277	0.651
SO_4_^2−^	0.990	0.001 **
K	0.183	0.768
Ca	0.899	0.038 *
Na	−0.139	0.823
Mg	0.919	0.027 *
Ba	0.540	0.347
Total P	0.113	0.857
Total S	0.958	0.010 *
Al	−0.158	0.800
Mn	−0.937	0.019 *
Co	0.221	0.721
Ni	0.735	0.157
Cu	−0.147	0.814
Zn	−0.228	0.713
Fe	−0.380	0.528
TOC	0.553	0.334

*: significant correlation (*p* < 0.05); **: significant correlation (*p* < 0.01).

## Data Availability

Data are contained within the article.

## Supplementary Materials

The following supporting information can be downloaded at https://www.mdpi.com/article/10.3390/antibiotics14111144/s1. Figure S1: Degradation kinetics constants (k_obs_) of PG hydrolysis at different pH levels in YSL water. HCl and NaOH (1 M) solutions were utilized to adjust the pH of YSL to 7.0, 7.5, 8.0, 8.5, and 9.0, respectively; Figure S2: 3D fluorescence spectra of five different surface waters, including XLL, JXR, NJU, YSL, and XWL. (a) Original samples (b) Samples filtrated by C column (c) Samples filtrated by Na column; Figure S3: Degradation kinetics constants of PG hydrolysis in YSL surface water with additional microbial organic matters including tryptophan, albumin, phenylalanine, cysteine and glucose at 2 mg L^−1^; Figure S4: Different attacking directions of PG hydrolysis (Alpha-side and Beta-side); Figure S5: Transition state of PG hydrolysis under different conditions; Figure S6: A site map of the sample collection subsection in XLL, JXR, NJU, YSL, and XWL. All samples in our experiment were collected on 14 April. The weather was sunlight at 20 °C, approximately. Table S1: Physicochemical parameters of five natural waters; Table S2: Physicochemical properties of five natural waters after C column and Na column treatment; Table S3: Hydrolysis products of PG degradation in natural waters.

## References

[1] Li D., Yang M., Hu J.Y., Zhang Y., Chang H., Jin F. (2008). Determination of penicillin G and its degradation products in a penicillin production wastewater treatment plant and the receiving river. Water Res..

[2] Raynor B.D. (1997). Penicillin and ampicillin. Prim. Care Update OB/GYNS.

[3] Kümmerer K. (2009). Antibiotics in the aquatic environment—A review—Part I. Chemosphere.

[4] Chen J., Sun P., Zhou X., Zhang Y., Huang C.H. (2015). Cu(II)–catalyzed transformation of benzylpenicillin revisited: The overlooked oxidation. Environ. Sci. Technol..

[5] Zhang Y., Xiao Y., Zhong Y., Lim T.T. (2019). Comparison of amoxicillin photodegradation in the UV/H_2_O_2_ and UV/persulfate systems: Reaction kinetics, degradation pathways, and antibacterial activity. Chem. Eng. J..

[6] Kümmerer K. (2009). Antibiotics in the aquatic environment—A review—Part II. Chemosphere.

[7] Chen J., Wang Y., Qian Y., Huang T. (2017). Fe(III)-promoted transformation of β-lactam antibiotics: Hydrolysis vs oxidation. J. Hazard. Mater..

[8] Walsh C. (2000). Molecular mechanisms that confer antibacterial drug resistance. Nature.

[9] Sui Q., Wang B., Zhao W.T., Huang J., Yu G., Deng S.B., Qiu Z.F., Lu S.G. (2012). Identification of priority pharmaceuticals in the water environment of China. Chemosphere.

[10] Leung H.W., Minh T.B., Murphy M.B., Lam James C.W., So M.K., Martin M., Lam Paul K.S., Richardson B.J. (2012). Distribution, fate and risk assessment of antibiotics in sewage treatment plants in Hong Kong, South China. Environ. Int..

[11] Hu Z., Periyannan G., Bennett B., Crowder M.W. (2008). Role of the Zn1 and Zn2 sites in Metallo-β-lactamase L1. J. Am. Chem. Soc..

[12] Kaminskaia N.V., Spingler B., Lippard S.J. (2000). Hydrolysis of β-lactam antibiotics catalyzed by dinuclear zinc(ii) complexes:  Functional mimics of metallo-β-lactamases. J. Am. Chem. Soc..

[13] Bahr G., González L.J., Vila A.J. (2021). Metallo-β-lactamases in the age of multidrug resistance: From structure and mechanism to evolution, dissemination, and inhibitor design. Chem. Rev..

[14] Mitchell S.M., Ullman J.L., Teel A.L., Watts R.J. (2014). pH and temperature effects on the hydrolysis of three β-lactam antibiotics: Ampicillin, cefalotin and cefoxitin. Sci. Total Environ..

[15] Mabey W., Mill T. (1978). Critical review of hydrolysis of organic compounds in water under environmental conditions. J. Phys. Chem. Ref. Data.

[16] Guo Y., Tsang D.C.W., Zhang X., Yang X. (2018). Cu(II)-catalyzed degradation of ampicillin: Effect of pH and dissolved oxygen. Environ. Sci. Pollut. Res..

[17] Gensmantel N.P., Proctor P., Page M.I. (1980). Metal-ion catalysed hydrolysis of some β-lactam antibiotics. J. Chem. Soc. Perkin Trans..

[18] Sheng F., Ling J.Y., Wang C., Jin X., Gu X.Y., Li H., Zhao J.T., Wang Y.J., Gu C. (2019). Rapid Hydrolysis of penicillin antibiotics mediated by adsorbed zinc on goethite surfaces. Environ. Sci. Technol..

[19] Jin X., Wu D.D., Ling J.Y., Wang C., Liu C., Gu C. (2019). Hydrolysis of chloramphenicol catalyzed by clay minerals under nonaqueous conditions. Environ. Sci. Technol..

[20] Hirte K., Seiwert B., Schüürmann G., Reemtsma T. (2016). New hydrolysis products of the beta-lactam antibiotic amoxicillin, their pH-dependent formation and search in municipal wastewater. Water Res..

[21] Chen J., Sun P., Zhang Y., Huang C.H. (2016). Multiple roles of Cu(II) in catalyzing hydrolysis and oxidation of β-lactam antibiotics. Environ. Sci. Technol..

[22] Huang T., Fang C., Qian Y., Gu H., Chen J. (2017). Insight into Mn(II)-mediated transformation of β-lactam antibiotics: The overlooked hydrolysis. Chem. Eng. J..

[23] Diaz N., Sordo T.L., Suarez D., Mendez R., Martin-Villacorta J. (2004). Zn^2+^ catalysed hydrolysis of β-lactams: Experimental and theoretical studies on the influence of the β-lactam structure. New J. Chem..

[24] Morris J.J., Page M.I. (1980). Intra- and inter-molecular catalysis in the aminolysis of benzylpenicillin. J. Chem. Soc. Perkin Trans..

[25] Llinás A., Donoso J., Vilanova B., Frau J., Muñoz F., Page M.I. (2000). Thiol-catalysed hydrolysis of benzylpenicillin. J. Chem. Soc. Perkin Trans..

[26] Jiang M., Wang L., Ji R. (2010). Biotic and abiotic degradation of four cephalosporin antibiotics in a lake surface water and sediment. Chemosphere.

[27] Xu H., Cooper W.J., Jung J., Song W. (2011). Photosensitized degradation of amoxicillin in natural organic matter isolate solutions. Water Res..

[28] Andreozzi R., Caprio V., Ciniglia C. (2004). Antibiotics in the environment:  Occurrence in Italian STPs, fate, and preliminary assessment on algal toxicity of amoxicillin. Environ. Sci. Technol..

[29] Timm A., Borowska E., Majewsky M., Merel S., Zwiener C., Baase S., Horn H. (2019). Photolysis of four β-lactam antibiotics under simulated environmental conditions: Degradation, transformation products and antibacterial activity. Sci. Total Environ..

[30] Alekseev V.G. (2012). Metal complexes of penicillins and cephalosporins (Review). Pharm. Chem. J..

[31] Wang H., Yao H., Sun P., Li D., Huang C.-H. (2016). Transformation of tetracycline antibiotics and Fe(II) and Fe(III) species induced by their complexation. Environ. Sci. Technol..

[32] Wang H., Yao H., Sun P.Z., Pei J., Li D.S., Huang Q.H. (2015). Oxidation of tetracycline antibiotics induced by Fe(III) ions without light irradiation. Chemosphere.

[33] Navarro P.G., Blazquez I.H., Osso B.Q., Parras P.J., Puentedura M.I.M., Garcia A.M. (2003). Penicillin degradation catalysed by Zn(II) ions in methanol. Int. J. Biol. Macromol..

[34] Her N., Amy G., McKnight D., Sohn J., Yoon Y. (2003). Characterization of DOM as a function of MW by fluorescence EEM and HPLC-SEC using UVA, DOC, and fluorescence detection. Water Res..

