# Supplementary material for: The Domination of Penicillin G Degradation in Natural Surface Water: Effect of Calcium Ion and Biological Dissolved Organic Matter
Source: Antibiotics (Basel). 2025 Nov 11;14(11):1144. doi: 10.3390/antibiotics14111144 (PMC12649314; doi:10.3390/antibiotics14111144)
Supplement: Supplementary file 1 [file antibiotics-14-01144-s001.zip › antibiotics-3916183-supplementary.pdf]

Supporting Information

The Domination of Penicillin G Degradation in Natural Surface Water: Effect of Calcium Ion and Biological Dissolved Organic Matter

Feng Sheng [1], Jingyi Ling [2,*], Na Mi [1], Jixing Wan [1], Lu Yang [1], Ming Li [1], Chao Wang [2] and Jiaqi Shi [1,*]

[1] Key Laboratory of Soil Environmental Management and Pollution Control, Nanjing Institute of Environmental Sciences, Ministry of Ecology and Environment, Nanjing 210042, China; shengfeng@nies.org (F.S.); mina@nies.org (N.M.); wanjixing@nies.org (J.W.); yanglu@nies.org (L.Y.); liming@nies.org (M.L.)

[2] Jiangsu Province Engineering Research Center of Synergistic Control of Pollution and Carbon Emissions in Key Industries, Jiangsu Provincial Environmental Engineering Technology Co., Ltd., Nanjing 210019, China; chaowang@nju.edu.cn

* Correspondence: lingjingyi@jsep.com (J.L.); sjq@nies.org (J.S.)

Content S1:

The hydrolysis and photolysis products of PG were determined by HPLC-QTOF-MS. An Atlantic T3 C18 column (Waters, 3 μm, 2.1 × 100 mm) was used and the mobile phase consisted of 0.1% formic acid (A) and methanol (B). The eluent gradient started with 80% A for 3 min; and decreased to 50% in 12 min, then continued to decrease to 10% in 5 min, finally returned to the initial condition within 2min for another 8 min. The column temperature and injection volume were 30 °C and 5 μL, respectively. The flow rate was set as 0.2 mL min^{-1}. MS analysis equipped with an electrospray ion source (ESI) was conducted in positive mode in the *m/z* ranged from 50 - 1000. Parameters setting for mass spectrometer were as followed: Ions source gas 1, 55 psi; ion source gas 2, 55 psi; temperature, 550 °C; ionspray voltage floating, 5500 V; curtain gas, 35 psi; declustering potential, 100 V; collision energy, 10 V. In the MS/MS analysis, collision energy switching (25 ± 5 V) was used to obtain an optimized dissociation. The data was analyzed by PeakView software (Version 1.2, AB Sciex).

Content S2:

The pH and conductivity of surface water were detected by pH meter (Thermal Scientific, USA). The concentrations of total S and P, and cations including K, Ca, Na, Mg, Ba, Al, Mn, Co, Ni, Cu, Zn and Fe ion were detected ICP-OES (Nexion 300X, PE, USA), while the concentrations of anions including Cl^-, NO_3^- and SO_4^{2-} were measured by IC (ICS900, Thermal Scientific, USA). Moreover, total organic carbon of surface water was also measured by TOC analyzer (TOC-5000A, Shimadzu, Japan).

Content S3:

Fluorescence excitation-emission matrix (EEM) was conducted in a FluoroMax-4 Spectrofluorometer (HORIBA Scientific, France). Maximum emission intensity was $2 \cdot 10^6$ CPU. The excitation wavelength ranged from 200 to 500 nm, and emission wavelength ranged from 250 to 550 nm with 5 nm steps. The obtained fluorescence spectrum was divided into five areas, including Region I (Excited Wavelength :200-250 nm; Emission Wavelength: 250-330 nm), II (Excited Wavelength :200-250 nm; Emission Wavelength: 330-380 nm), III (Excited wavelength :200-250 nm; Emission Wavelength: 380-550 nm), IV (Excited Wavelength: 250-490 nm; Emission Wavelength: 250-380 nm), V (Excited Wavelength: 250-490 nm; Emission Wavelength: 380-550 nm), which referred to the simple aromatic proteins, simple aromatic proteins, fulvic acid-like materials, soluble microbial byproduct-like materials and humic acid-like organics, respectively. The obtained data were conducted in MATLAB R2018 to eliminate the disturbance of Rayleigh scattering.

Table S1 Physicochemical parameters of five natural waters

Parameter	XLL	JXR	NJU	YSL	XWL
pH	7.95	7.80	8.09	8.14	8.52
Conductivity(μS/cm)	556	537	436	586	291
Cl^- (mg L^{-1})	11.6	35.2	24.8	10.0	15.4
NO_3^- (mg L^{-1})	1.10	5.27	1.86	0.64	2.26
SO_4^{2-} (mg L^{-1})	96.2	74.2	52.01	146	30.3
K (mg L^{-1})	4.44	8.11	4.15	4.61	4.16
Ca (mg L^{-1})	99.9	66.0	66.4	104	40.7
Na (mg L^{-1})	18.0	35.4	23.1	14.6	16.7
Mg (mg L^{-1})	18.2	12.9	10.05	18.5	7.95
Ba (mg L^{-1})	0.07	0.05	0.07	0.08	0.06
Total P (mg L^{-1})	0.03	0.06	0.01	0.01	0.02
Total S (mg L^{-1})	46.6	27.9	22.3	58.7	12.7
Al (μg L^{-1})	7.05	7.49	135	17.0	215
Mn (μg L^{-1})	4.41	8.08	11.1	3.59	11.8
Co (μg L^{-1})	0.24	0.29	0.22	0.20	0.19
Ni (μg L^{-1})	3.90	3.58	2.64	3.30	1.79
Cu (μg L^{-1})	1.26	6.27	1.92	0.85	2.00
Zn (μg L^{-1})	4.12	14.6	5.80	2.75	6.23
Cd (μg L^{-1})	0.00	0.01	0.01	0.00	0.01
Fe (μg L^{-1})	6.50	7.20	1869	34.9	17.9
TOC (mg L^{-1})	7.01	7.25	5.76	6.07	4.77

Table S2 Physicochemical properties of five natural waters after C column and Na column treatment

Locations	Na column (mg L^{-1})			C column (mg L^{-1})		
	Ca^{2+}	Mg^{2+}	K^+	Ca^{2+}	Mg^{2+}	K^+
XLL	1.33	1.98	6.16	71.28	3.15	18.62
JXR	ND	0.39	ND	60.57	6.59	12.97
NJU	ND	0.47	0.15	61.21	3.01	9.98
YSL	58.84	3.38	29.06	88.25	1.82	18.32
XWL	ND	0.32	ND	37.96	3.04	7.86

ND means not detected. The other metals (Ba, Al, Mn, Co, Ni, Cu, Zn, Cd and Fe) were also detected and the concentration of those were all below the detection limits.

Table S3 Hydrolysis products of PG degradation in natural waters

TP Number	Product Name	RT (min)	Formula	m/z (Meausured)	m/z (Theoretical)
0	Penicillin G	20.02	$C_{16}H_{18}N_2O_4S$	335.1052	335.11
1	Penicilloic acid	17.79;18.02	$C_{16}H_{20}N_2O_5S$	353.1159	353.11
2	Benzylpenilloic acid	17.29;17.53	$C_{15}H_{20}N_2O_3S$	309.1257	309.13
3	Phenylacetic acid	—	$C_8H_8O_2$	—	—
4	6-Aminopenicillanic acid	3.13;13.90	$C_8H_{12}N_2O_3S$	217.1035	217.17
5	Product 5	22.83	$C_8H_{14}N_2O_4S$	235.1294	235.08
6	Product 6	24.36	$C_6H_{11}NO_2S$	162.0950	162.06

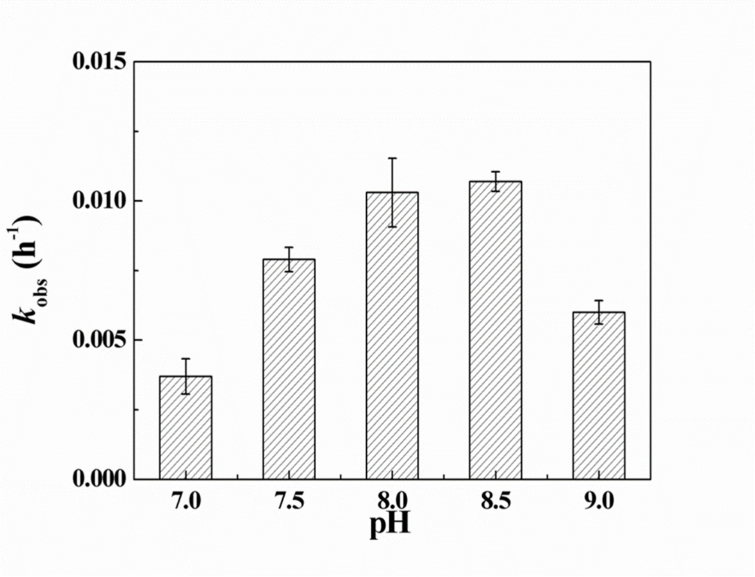

Figure S1 Degradation kinetics constants (k_{obs}) of PG hydrolysis at different pH levels in YSL water. HCl and NaOH (1 M) solutions were utilized to adjust the pH of YSL to 7.0, 7.5, 8.0, 8.5 and 9.0, respectively.

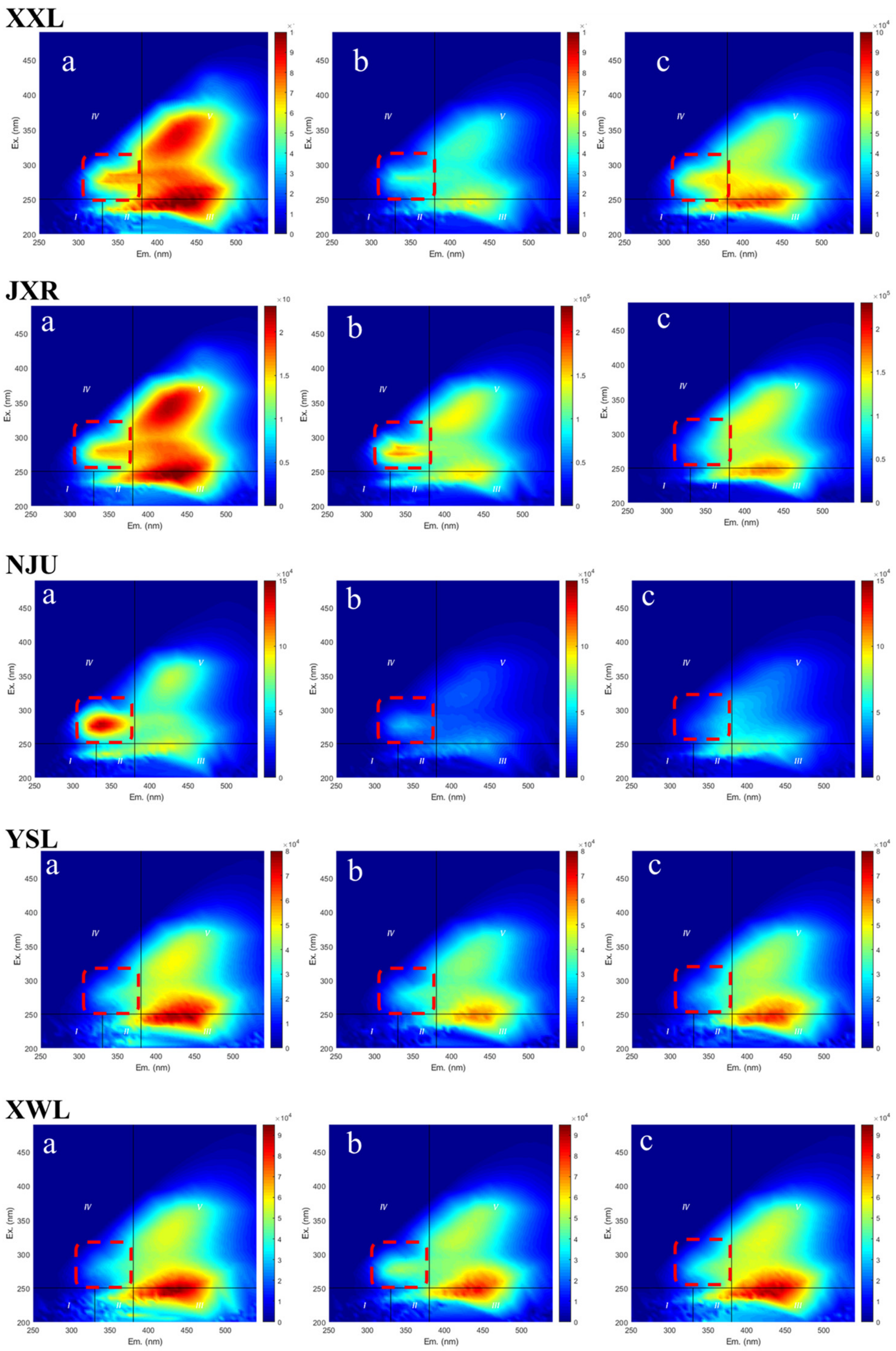

Figure S2 3D fluorescence spectra of five different surface waters including XLL, JXR, NJU, YSL and XWL. (a) Original samples (b) Samples filtrated by C column (c) Samples filtrated by Na column.

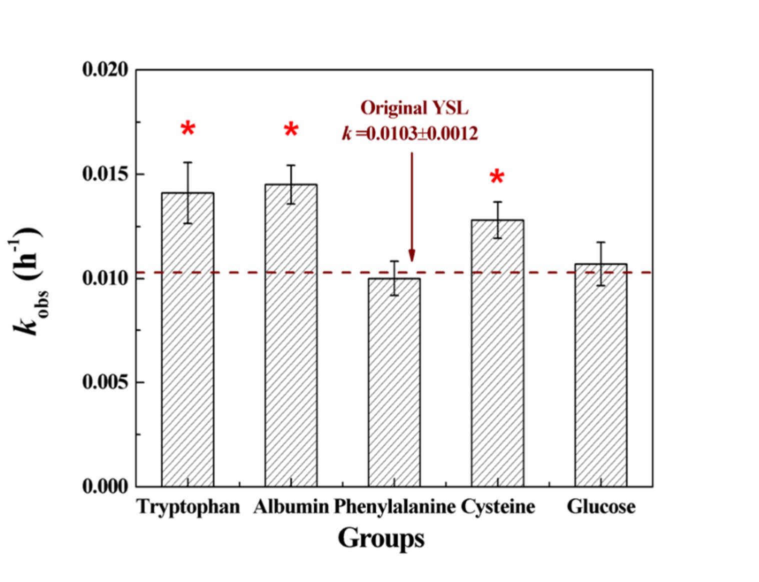

Figure S3 Degradation kinetics constants of PG hydrolysis in YSL surface water with additional microbial organic matters including tryptophan, albumin, phenylalanine, cysteine and glucose at 2 mg L^{-1}. “*” means a significant correlation ($p<0.05$) against other groups.

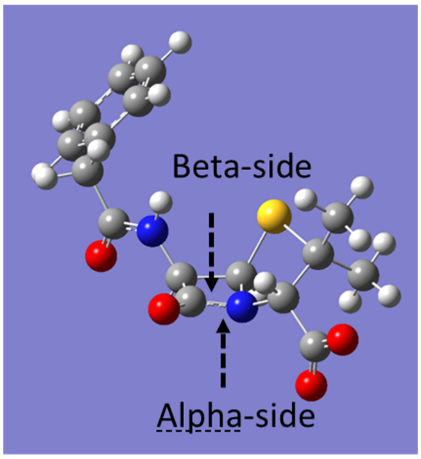

Figure S4 Different attacking directions of PG hydrolysis (Alpha-side and Beta-side)

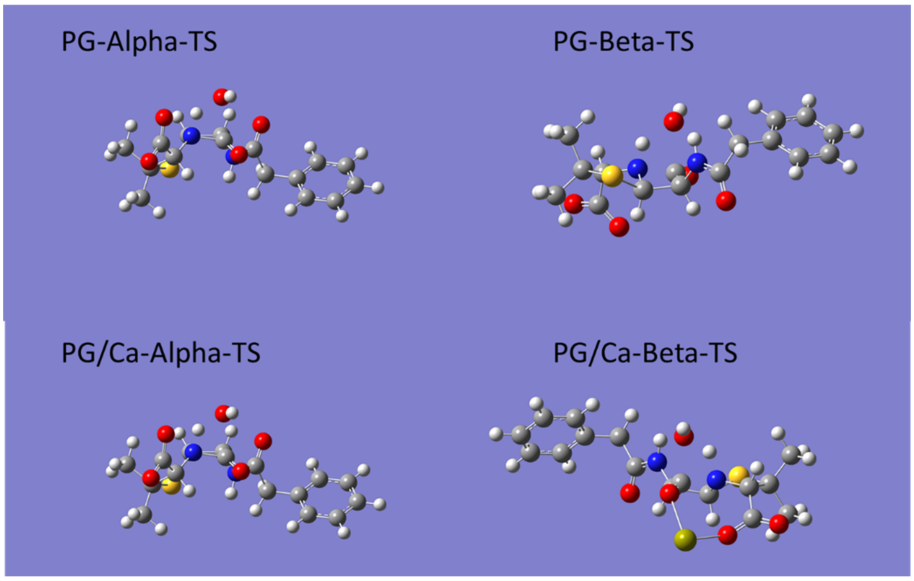

Figure S5 Transition state of PG hydrolysis under different conditions

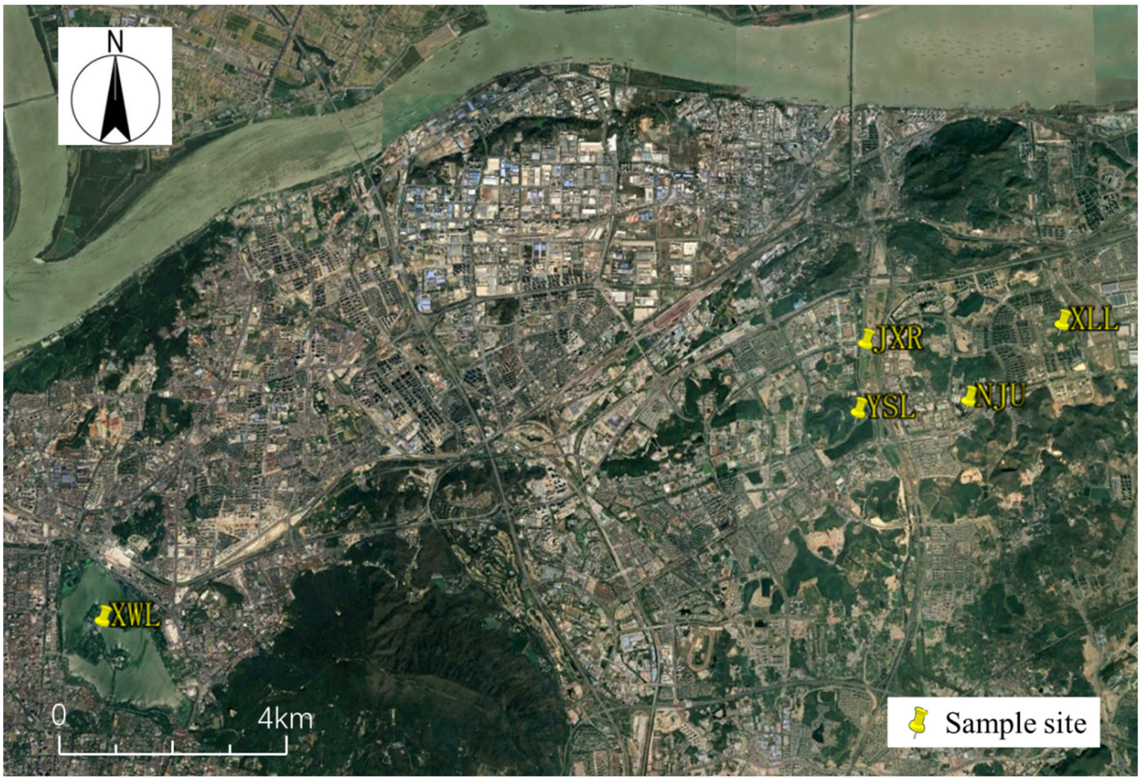

Figure S6 A site map of sample collection subsection in XLL, JXR, NJU, YSL and XWL. All samples in our experiment were collected on 14 April. The weather was sunlight at 20℃, approximately.
